# Superficial Circumflex Iliac Artery Free Flap for Coverage of Hand Injuries

**DOI:** 10.7759/cureus.31520

**Published:** 2022-11-15

**Authors:** Touqeer Hussain, Fahad H Khan, Obaid ur Rahman, Mirza Shehab A Beg

**Affiliations:** 1 Plastic and Reconstructive Surgery, Liaquat National Hospital and Medical College, Karachi, PAK

**Keywords:** microsurgery, free groin flap, scia flap, free flaps, hand defects, hand injuries

## Abstract

Open de-gloving hand injuries with exposed tendons and bones require coverage by a flap. Conventionally used groin or abdominal flaps are cumbersome to patients due to extensive dressing and prolonged passive positioning of the hand until pedicle division. Superficial circumflex iliac artery (SCIA) flap is evolved from a traditional groin flap, and because of its thinness, pliability, and concealed donor site, it is an ideal option for single-stage reconstruction of traumatic hand defects avoiding discomforting passive hand position, joint stiffness, and unexpected flap avulsion which were associated with traditional groin flap. All patients with exposed bones or tendons due to traumatic hand injuries who opted for free flap coverage during the year 2018 to 2020 were enrolled in our study. After initial debridement, the wound was covered with a free SCIA flap. Duration of hospital stay, days out of work, the number of dressings required, postoperative complications, and any secondary procedures for flap readjustment were noted till six months postoperatively. A total of eight patients were included in the study. The mechanism of injury was road traffic accidents in a single patient and occupational injury in eight patients. The average duration of hospital stay was six days after reconstructive surgery. The average number of dressings a patient had was 18, and only two patients required flap thinning. Only one patient had a postoperative infection which was managed with dressings and antibiotics. One patient had peripheral flap necrosis. We had zero flap re-exploration. Therefore, we conclude that hand defects coverage with SCIA flap leads to a smaller number of working days lost and rarely requires secondary procedures.

## Introduction

Traumatic hand injuries are common worldwide, and most of the global burden of complex hand injuries requiring surgical intervention is shared by South and East Asia [1]. Most hand injuries are occupational, occur in the dominant hand, and result in fractures of small bones, tendon injuries, and soft tissue loss [2,3]. De-gloving hand injuries require coverage by fasciocutaneous flap. Skin grafts (both split skin graft [SSG] and full-thickness skin graft [FTSG]) are not a good option for coverage in traumatic hand injuries either due to exposed bones, exposed tendons, or the need for secondary tendon reconstruction [4]. Preferred flaps for soft tissue coverage of hands, both palmar and dorsal aspects, are those which are soft, thin, and pliable and should have a better color match as hands are exposed areas, therefore aesthetically important [5].

Conventionally used flaps for hand wound coverage are pedicled groin flap or pedicled abdominal flap, depending on the size of the defect. Both above-mentioned flaps are two-staged and require pedicle division after at least three weeks [6,7].

Single-stage reconstruction of hand defects with free flaps is safe and reliable. Multiple options are available, including dorsalis pedis artery flap, scapular and parascapular flap, lateral arm flap, anterolateral thigh flap, and superficial circumflex iliac artery flap [8,9]. Advantages of reconstruction with a free flap include a single-stage procedure, availability of a large amount of tissue, and multiple choices to better match color and tissue thickness [10].

Superficial circumflex iliac artery (SCIA) flap is evolved form of a traditional groin flap; because of its thinness, pliability, and the concealed donor site is an ideal option for single-stage reconstruction of traumatic hand defects obscuring the need for discomforting passive hand position, joint stiffness and unexpected flap avulsion which were associated with traditional groin flap [11,12].

Our study aims to show the effectiveness of the superficial circumflex iliac artery flap (SCIA flap) for the coverage of hand defects resulting from traumatic injuries and to know its short-term complications.​​

## Materials and methods

It is a prospective descriptive study performed from 2018 to 2020 at Liaquat National Hospital, Karachi. After taking approval from the institutional review board and ethical committee, all patients with exposed bones or tendons due to traumatic hand injuries who opted for free flap coverage during these years were enrolled in our study after their informed consent.

After initial debridement, the wound was covered with a free SCIA flap. Demographic and clinical data, including age, gender, mechanism of injury, defect size, size of flap harvested, pedicle length, recipient vessels, and anastomosis detail, was collected. Postoperative duration of hospital stays, days out of work, the number of dressings changed, postoperative complications, and any secondary procedures for flap readjustment were noted.

Patients were followed for a minimum of six months postoperatively.

Surgical technique

All the surgeries were performed in a single stage. We started the surgery first by preparing the donor site. All the necrotic tissues of the injured hand were derided and made sure that the recipient bed was infection-free. Allen's test was performed to see the patency of both radial and ulnar arteries. Either radial or ulnar artery, depending upon the proximity to the wound, was explored and prepared for anastomosis along with one of its venae comitantes.

**Figure 1:**
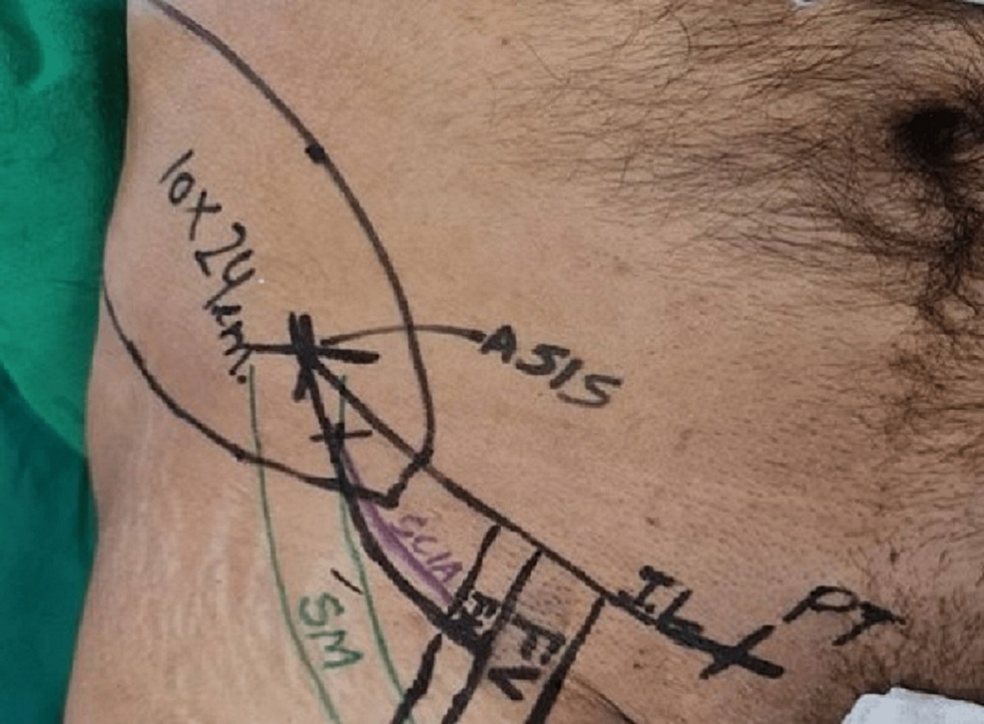
Identification and marking of pubic tubercle, anterior superior iliac spine, inguinal ligament, femoral artery, and sartorius muscle Markings of the flap start with identifying the pubic tubercle and ASIS. Sartorius muscle was marked from ASIS and the femoral artery and femoral vein were palpated and marked. The flap was marked lateral to ASIS. PT - pubic tubercle; ASIS - anterior superior iliac spine; IL - inguinal ligament; SM - sartorius muscle; FA - femoral artery; FV - femoral vein; SCIA - superficial circumflex iliac artery

A sandbag was placed beneath the pelvic bone for better exposure to the surgical field. Pre-operative Doppler examination to delineate the surgical anatomy of the superficial circumflex artery was crucial as SCIA was absent in 2% of the subjects. The pubic tubercle, anterior superior iliac spine, inguinal ligament, femoral artery, and sartorius muscle were all identified and marked (Figure 1).

**Figure 2:**
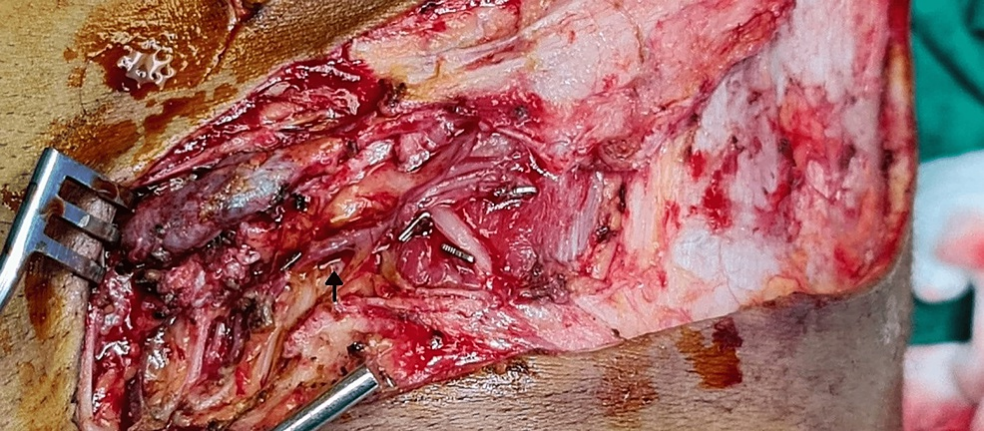
Superficial circumflex artery (arrow) as it arises from the medial border of sartorius

Artery usually runs two fingerbreadths below and parallel to the inguinal ligament and was identified using handheld Doppler and marked. The skin paddle was designed lateral to pubic tubercle keeping SCIA at the center of the transverse axis of the flap. The lateral edge of the flap can be extended beyond the anterior superior iliac spine. The lateral incision was made first deep till deep fascia. The flap was elevated above the deep fascia. Dissection was done from the lateral to medial direction until lateral border of the sartorius was identified. The dissection plane was then deepened deep to the deep fascia. Dissection continued in the deep plane until the pedicle was visualized emerging along the medial border of the sartorius (Figure 2).

Then medial skin incision was completed, and the pedicle was skeletonized and dissected up to its origin from the femoral artery.

The flap was divided, the donor site was closed primarily, and after the flap was inset on the recipient site, anastomosis was done with an 8/0 polypropylene suture.

Postoperatively intravenous heparin was given for anticoagulation, and five days course of ceftazidime antibiotic was given to all patients. Patients were mobilized out of bed on the first postoperative day.

**Figure 3:**
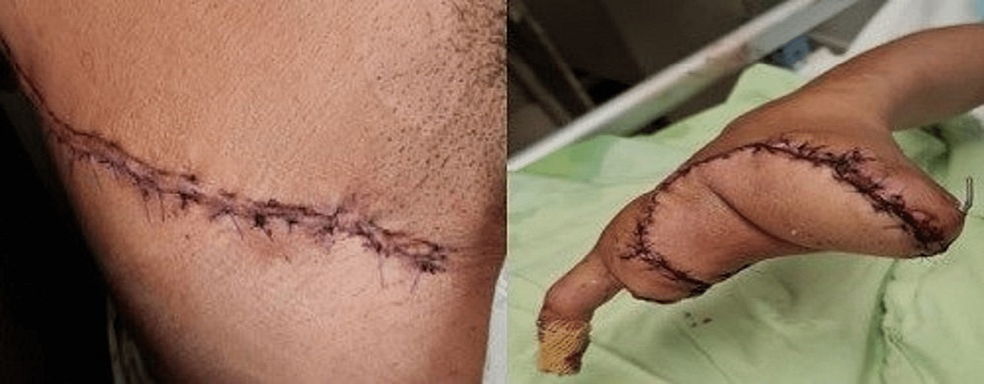
Primarily closed donor site (left) with well-vascularized and contoured flap on the fifth postoperative day

Postoperatively flap was monitored hourly in the first 24 hours, then every two hours till the fourth postoperative day by analyzing flap color, turgor, capillary refill, and needle prick (Figure 3). 

## Results

A total of eight patients were operated on in the selected years. Six of them were males, and two were females. The average age was 28±1.75 years. Mechanism of injury was a road traffic accident in a single patient and occupational injury in all others. Dominant hand was affected in six patients and non-dominant in two. The mean defect size and mean dimensions of harvested flaps were 10.2x6.5 cm with donor site primary closure. The average duration of hospital stay was five days after reconstructive surgery. The average number of postoperative dressings a patient had until the wound fully healed was 18, and only two patients required flap thinning due to bulky groin. One patient had a postoperative infection with purulent discharge from the wound bed, which was managed conservatively with dressings, wound irrigation, and antibiotics. One patient had peripheral flap necrosis, which healed with secondary intention. We had zero flap re-exploration. Demographic data is summarized in Table 1, and early postoperative complications are shown in Table 2. 

**Table 1:** Demographic data

Patient characteristics		No of patients (n)
Total patients		8
Male		6
Female		2
Mean age of patients		28 ± 1.75yrs
Mechanism of injury	Road traffic accident	1
	Occupational injury	7
Dominant hand affected		6
Mean duration of hospital stays		5 days

**Table 2:** Early postoperative complications

Complications	No of patients (n)
Flap re-exploration	0
Total flap failure	0
Partial flap necrosis	1
Wound infection	1
Wound dehiscence	0
Donor site collection	0
Donor site infection	0
Donor site dehiscence	0

## Discussion

Coverage of traumatic hand defects remains a dare for reconstructive surgeons due to the key input of hands in daily living activities, small joints need to remain supple during the reconstruction process to continue function, and web spaces between each digit should be recreated at the end of the reconstructive process [13,14]. All of the above requirements are met only with thin and pliable flaps [8].

Conventionally used flaps for hand reconstruction are pedicled groin flap or pedicled abdominal flap. The pedicled groin is a workhorse flap for hand reconstruction in the plastic surgeons' armature because of its malleability and slimness. Donor sites can be primarily closed even with extended groin flaps, which can be harvested beyond the midaxillary line starting at the lateral border of the sartorius. The trouble with the pedicled groin is that the recipient area (hand) remains attached to the donor area (groin) for at least three weeks which is quite troublesome to patients. The patient needs extensive dressings for three weeks, and the inability to perform physiotherapy during these three weeks leads to stiffness of small hand joints adding morbidity to the patient. Furthermore, it requires additional surgery of pedicle division [15]. Similarly, pedicled abdominal flaps based on periumbilical perforators provide extensive soft tissue coverage for hand degloving injuries, but they are quite bulky due to the thick layer of fat both above and below the camper's fascia. The abdominal flap also requires pedicle division after three weeks and an additional procedure of flap thinning months after the flap take [7,16].

Another pedicle flap option for hand reconstruction is the reverse radial forearm flap which is single-stage and with excellent color and texture match, but this flap is more prone to venous congestion due to reversal in the direction of venous outflow after flap inset, and the donor site needs graft on exposed volar forearm [17,18].

Microvascular reconstruction of hand defects with a free superficial circumflex iliac perforator flap is a single-stage straightforward option that provides smooth coverage with various advantages.

Wen-Ming et al. [19] successfully performed SCIA flap in 12 subjects to cover a variety of recipient sites. This flap not only overcomes most of the drawbacks of the free groin flap but also exhibits many of its advantages, including an inconspicuous, primarily closed donor site that seldom requires skin grafting, the availability of a large hairless, thin, non-bulky skin flap with longer arterial pedicle (3 to 13 cm in their cases). In addition to the above-mentioned advantages, we also found that the SCIA flap requires less number of dressings change as compared to the pedicled groin flap, early return to work, and more favorable postoperative joints condition due to ongoing physiotherapy in the recovery period, which was heralded in conventional groin flap till pedicle division [19].

Altiparmark et al. [20] performed a meta-analysis of 36 articles, including 907 superficial circumflex artery perforator flaps. They labeled this flap as a workhorse flap of reconstructive surgery due to its long pedicle length of 5 cm and low failure and complication rates (2.7% of failure rate and 4.2% of complication rate) in contrast to our study, which has zero failure rate and 25% complication rate, but our complications were minor and easily managed conservatively. The most frequent causes of defects were tumors (38.2%), and lower extremities were the most common recipient areas (62.7%) in the conducted meta-analysis. However, in our study, we stick to a single recipient site, i.e., hand, and single etiology, i.e., trauma.

Kwon et al. [21] made some modifications in the dissection of the SCIA flap by marking the medial border of the flap away from the pubic tubercle and dissecting the flap superficial to superficial fascia, thereby obtaining a good pedicle length of 6 to 8 cm. Still, his modification requires preoperative angiogram which is not cost-effective in our setup, and we got the desired pedicle length by traditional SCIA flap, and none of our anastomosis required intervening vein graft.

There are certain limitations to our study; it is an observational study, not a comparative one; therefore efficacy of free SCIA flap is not compared with other traditionally in-use flaps. Furthermore, only short-term flap-related complications were observed in our study, and long-term patient functional outcome was not observed.

## Conclusions

Hand defects coverage with SCIA flap leads to a smaller number of working days lost and rarely leads to any short-term complications, as shown by our results. It is thin and pliable and gives excellent contour to working hands so that there is little hindrance in return to occupation. We strongly recommend free SCIA flap for hand defects coverage in centers where good microvascular expertise is available.

## References

[1] Crowe CS, Massenburg BB, Morrison SD (2020). Global trends of hand and wrist trauma: a systematic analysis of fracture and digit amputation using the Global Burden of Disease 2017 Study. Inj Prev.

[2] Gyer G, Michael J, Inklebarger J (2018). Occupational hand injuries: a current review of the prevalence and proposed prevention strategies for physical therapists and similar healthcare professionals. J Integr Med.

[3] Bhatti DS, Ain NU, Fatima M (2020). Occupational hand-related injuries at a major tertiary care burn and reconstructive center in Pakistan. Cureus.

[4] Goel P, Badash I, Gould DJ, Landau MJ, Carey JN (2018). Options for covering large soft tissue defects in the setting of trauma. Current Trauma Reports.

[5] Naalla R, Chauhan S, Dave A, Singhal M (2018). Reconstruction of post-traumatic upper extremity soft tissue defects with pedicled flaps: An algorithmic approach to clinical decision making. Chin J Traumatol.

[6] Acharya AM, Ravikiran N, Jayakrishnan KN, Bhat AK (2019). The role of pedicled abdominal flaps in hand and forearm composite tissue injuries: Results of technical refinements for safe harvest. J Orthop.

[7] Al-Qattan MM, Al-Qattan AM (2016). Defining the indications of pedicled groin and abdominal flaps in hand reconstruction in the current microsurgery era. J Hand Surg Am.

[8] Das De S, Sebastin SJ (2019). Considerations in flap selection for soft tissue defects of the hand. Clin Plast Surg.

[9] Benanti E, De Santis G, Leti Acciaro A, Colzani G, Baccarani A, Starnoni M (2020). Soft tissue coverage of the upper limb: a flap reconstruction overview. Ann Med Surg.

[10] Ono S, Sebastin SJ, Ohi H, Chung KC (2017). Microsurgical flaps in repair and reconstruction of the hand. Hand Clin.

[11] Pan Z, Jiang P, Xue S, Zhao Y, Li H, Gao P, Wang J (2020). Use of free sensate SCIA flap for reconstruction of distal limb defects of moderate size. J Plast Reconstr Aesthet Surg.

[12] Wang HD, Alonso-Escalante JC, Cho BH, DeJesus RA (2017). Versatility of free cutaneous flaps for upper extremity soft tissue reconstruction. J Hand Microsurg.

[13] Bashir MM, Sohail M, Shami HB (2018). Traumatic wounds of the upper extremity: coverage strategies. Hand Clin.

[14] Starnoni M, Benanti E, Acciaro AL, De Santis G (2021). Upper limb traumatic injuries: a concise overview of reconstructive options. Ann Med Surg.

[15] Amouzou KS, Berny N, El Harti A, Diouri M, Chlihi A, Ezzoubi M (2017). The pedicled groin flap in resurfacing hand burn scar release and other injuries: a five-case series report and review of the literature. Ann Burns Fire Disasters.

[16] Jabaiti S, Ahmad M, AlRyalat SA (2020). Reconstruction of upper extremity defects by random pedicle abdominal flaps: is it still a valid option?. Plast Reconstr Surg Glob Open.

[17] Akdag O, Karamese M, Selimoglu MN (2016). Reverse adipofascial radial forearm flap surgery for soft-tissue reconstruction of hand defects. Eplasty.

[18] Maan ZN, Legrand A, Long C, Chang JC (2017). Reverse radial forearm flap. Plast Reconstr Surg Glob Open.

[19] Hsu WM, Chao WN, Yang C, Fang CL, Huang KF, Lin YS, Lee TH (2007). Evolution of the free groin flap: the superficial circumflex iliac artery perforator flap. Plast Reconstr Surg.

[20] Altiparmak M, Cha HG, Hong JP, Suh HP (2020). Superficial circumflex iliac artery perforator flap as a workhorse flap: systematic review and meta-analysis. J Reconstr Microsurg.

[21] Kwon JG, Pereira N, Tonaree W, Brown E, Hong JP, Suh HP (2021). Long pedicled superficial circumflex iliac artery flap based on a medial superficial branch. Plast Reconstr Surg.

